# 1-(3-Chloro­phen­yl)thio­urea

**DOI:** 10.1107/S160053681203084X

**Published:** 2012-07-10

**Authors:** Hoong-Kun Fun, Ching Kheng Quah, Prakash S. Nayak, B. Narayana, B. K. Sarojini

**Affiliations:** a X-ray Crystallography Unit, School of Physics, Universiti Sains Malaysia, 11800 USM, Penang, Malaysia; b Department of Studies in Chemistry, Mangalore University, Mangalagangotri 574 199, India; c Department of Chemistry, P. A. College of Engineering, Nadupadavu, Mangalore 574 153, India

## Abstract

In the title compound, C_7_H_7_ClN_2_S, the thio­urea N—C(=S)—N plane forms a dihedral angle of 64.80 (6)° with the benzene ring. In the crystal, mol­ecules are linked *via* inter­molecular N—H⋯S and N—H⋯Cl hydrogen bonds into a sheet extending parallel to the (101) plane.

## Related literature

For related structures, see: Saleem & Yamin (2010 ▶); Sarojini *et al.* (2007 ▶). For standard bond-length data, see: Allen *et al.* (1987 ▶). For the stability of the temperature controller used for the data collection, see: Cosier & Glazer (1986 ▶).
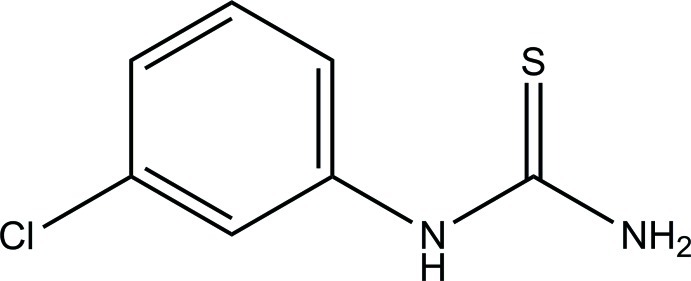


## Experimental

### 

#### Crystal data

C_7_H_7_ClN_2_S
*M* *_r_* = 186.66
Triclinic, 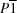

*a* = 5.4406 (3) Å
*b* = 8.5715 (4) Å
*c* = 9.2392 (4) Å
α = 104.221 (2)°
β = 91.776 (2)°
γ = 96.362 (2)°
*V* = 414.33 (3) Å^3^
*Z* = 2
Mo *K*α radiation
μ = 0.64 mm^−1^
*T* = 100 K
0.38 × 0.30 × 0.07 mm

#### Data collection

Bruker SMART APEXII DUO CCD area-detector diffractometer
Absorption correction: multi-scan (*SADABS*; Bruker, 2009 ▶) *T*_min_ = 0.791, *T*_max_ = 0.956
8525 measured reflections
2414 independent reflections
2194 reflections with *I* > 2σ(*I*)
*R*_int_ = 0.033

#### Refinement

*R*[*F* ^2^ > 2σ(*F* ^2^)] = 0.031
*wR*(*F* ^2^) = 0.085
*S* = 1.08
2414 reflections
112 parameters
H atoms treated by a mixture of independent and constrained refinement
Δρ_max_ = 0.68 e Å^−3^
Δρ_min_ = −0.37 e Å^−3^

### 

Data collection: *APEX2* (Bruker, 2009 ▶); cell refinement: *SAINT* (Bruker, 2009 ▶); data reduction: *SAINT*; program(s) used to solve structure: *SHELXTL* (Sheldrick, 2008 ▶); program(s) used to refine structure: *SHELXTL*; molecular graphics: *SHELXTL*; software used to prepare material for publication: *SHELXTL* and *PLATON* (Spek, 2009 ▶).

## Supplementary Material

Crystal structure: contains datablock(s) global, I. DOI: 10.1107/S160053681203084X/is5164sup1.cif

Structure factors: contains datablock(s) I. DOI: 10.1107/S160053681203084X/is5164Isup2.hkl

Supplementary material file. DOI: 10.1107/S160053681203084X/is5164Isup3.cml

Additional supplementary materials:  crystallographic information; 3D view; checkCIF report

## Figures and Tables

**Table 1:** Hydrogen-bond geometry (Å, °)

*D*—H⋯*A*	*D*—H	H⋯*A*	*D*⋯*A*	*D*—H⋯*A*
N2—H2N2⋯Cl1^i^	0.80 (2)	2.64 (2)	3.3583 (12)	150 (2)
N2—H1N2⋯S1^ii^	0.83 (3)	2.54 (3)	3.3619 (13)	167.5 (19)
N1—H1N1⋯S1^iii^	0.84 (2)	2.49 (3)	3.3149 (12)	167 (2)

Symmetry codes: (i) 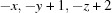; (ii) 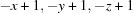; (iii) 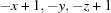.

**Fig. 1.**
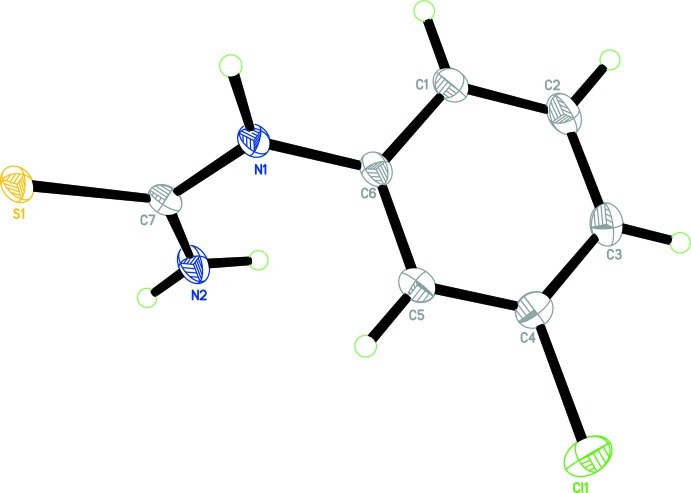
The molecular structure of the title compound showing 50% probability displacement ellipsoids for non-H atoms.

**Fig. 2.**
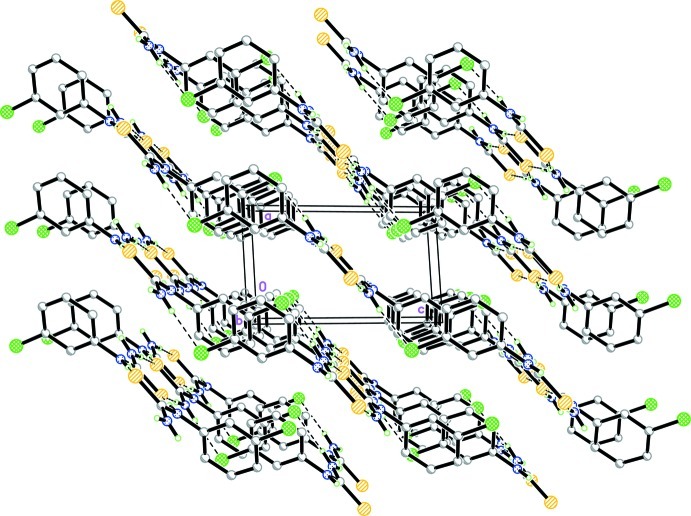
The crystal structure of the title compound, viewed along the b axis. H atoms not involved in hydrogen bonds (dashed lines) have been omitted for clarity.

## supplementary crystallographic information

### Comment

In view of importance of thiourea derivatives (Saleem & Yamin, 2010; Sarojini *et al.*, 2007), the title compound (I) is prepared and its crystal structure is reported.

In the title molecule (Fig. 1), the thiourea moiety (S1/N1/N2/C7) is planar (r.m.s. deviation = < 0.001) and it forms a dihedral angle of 64.80 (6)° with the benzene ring (C1–C6). Bond lengths (Allen *et al.*, 1987) and angles are within normal ranges. In the crystal structure (Fig. 2), molecules are linked *via* intermolecular N2—H2N2···S1, N2—H1N2···Cl1 and N1—H11···S1 hydrogen bonds (Table 1) into two-dimensional sheets parallel to the (101) plane.

### Experimental

3-Chloroaniline (0.65 ml, 0.0081 mol) was refluxed with potassium thiocyanate (1.4 g, 0.0142 mol) in 20 ml of water and 1.6 ml of conc. HCl for 3 h. The reaction mixture was then cooled to room temperature and stirred overnight. The precipitated product was then filetred, washed with water, dried and single crystals were grown from toluene and acetone (1:1) mixture by the slow evaporation method (*m.p.* 402 K).

### Refinement

N-bound hydrogen atoms were located in a difference Fourier map and refined freely [N—H = 0.80 (2)–0.84 (2) Å]. The remaining H atoms were positioned geometrically and refined using a riding model with C—H = 0.93 Å and *U*_iso_(H) = 1.2*U*_eq_(C).

### Crystal data

**Table:**

C_7_H_7_ClN_2_S	*Z* = 2
*M**_r_* = 186.66	*F*(000) = 192
Triclinic, *P*1	*D*_x_ = 1.496 Mg m^−^^3^
Hall symbol: -P 1	Mo *K*α radiation, λ = 0.71073 Å
*a* = 5.4406 (3) Å	Cell parameters from 5475 reflections
*b* = 8.5715 (4) Å	θ = 2.9–30.0°
*c* = 9.2392 (4) Å	µ = 0.64 mm^−^^1^
α = 104.221 (2)°	*T* = 100 K
β = 91.776 (2)°	Plate, colourless
γ = 96.362 (2)°	0.38 × 0.30 × 0.07 mm
*V* = 414.33 (3) Å^3^	

### Data collection

**Table:**

Bruker SMART APEXII DUO CCD area-detector diffractometer	2414 independent reflections
Radiation source: fine-focus sealed tube	2194 reflections with *I* > 2σ(*I*)
Graphite monochromator	*R*_int_ = 0.033
φ and ω scans	θ_max_ = 30.1°, θ_min_ = 2.3°
Absorption correction: multi-scan (*SADABS*; Bruker, 2009)	*h* = −7→7
*T*_min_ = 0.791, *T*_max_ = 0.956	*k* = −12→12
8525 measured reflections	*l* = −13→13

### Refinement

**Table:**

Refinement on *F*^2^	Primary atom site location: structure-invariant direct methods
Least-squares matrix: full	Secondary atom site location: difference Fourier map
*R*[*F*^2^ > 2σ(*F*^2^)] = 0.031	Hydrogen site location: inferred from neighbouring sites
*wR*(*F*^2^) = 0.085	H atoms treated by a mixture of independent and constrained refinement
*S* = 1.08	*w* = 1/[σ^2^(*F*_o_^2^) + (0.0374*P*)^2^ + 0.222*P*] where *P* = (*F*_o_^2^ + 2*F*_c_^2^)/3
2414 reflections	(Δ/σ)_max_ = 0.001
112 parameters	Δρ_max_ = 0.68 e Å^−^^3^
0 restraints	Δρ_min_ = −0.37 e Å^−^^3^

### Special details

**Table:**

Experimental. The crystal was placed in the cold stream of an Oxford Cryosystems Cobra open-flow nitrogen cryostat (Cosier & Glazer, 1986) operating at 100.0 (1) K.
Geometry. All esds (except the esd in the dihedral angle between two l.s. planes) are estimated using the full covariance matrix. The cell esds are taken into account individually in the estimation of esds in distances, angles and torsion angles; correlations between esds in cell parameters are only used when they are defined by crystal symmetry. An approximate (isotropic) treatment of cell esds is used for estimating esds involving l.s. planes.
Refinement. Refinement of F^2^ against ALL reflections. The weighted R-factor wR and goodness of fit S are based on F^2^, conventional R-factors R are based on F, with F set to zero for negative F^2^. The threshold expression of F^2^ > 2sigma(F^2^) is used only for calculating R-factors(gt) etc. and is not relevant to the choice of reflections for refinement. R-factors based on F^2^ are statistically about twice as large as those based on F, and R- factors based on ALL data will be even larger.

### Fractional atomic coordinates and isotropic or equivalent isotropic displacement parameters (Å^2^)

**Table:**

	*x*	*y*	*z*	*U*_iso_*/*U*_eq_	
Cl1	0.18973 (6)	0.42500 (4)	1.19169 (4)	0.02562 (10)	
S1	0.60726 (6)	0.24290 (3)	0.46595 (4)	0.01698 (10)	
N1	0.2888 (2)	0.14096 (13)	0.64519 (13)	0.0164 (2)	
N2	0.2660 (2)	0.39857 (14)	0.61960 (14)	0.0190 (2)	
C1	−0.0855 (2)	0.05999 (16)	0.76120 (15)	0.0181 (2)	
H1	−0.1397	−0.0171	0.6730	0.022*	
C2	−0.2277 (2)	0.07757 (17)	0.88598 (16)	0.0214 (3)	
H2	−0.3772	0.0116	0.8802	0.026*	
C3	−0.1494 (2)	0.19203 (17)	1.01876 (15)	0.0201 (3)	
H3	−0.2460	0.2046	1.1012	0.024*	
C4	0.0770 (2)	0.28730 (15)	1.02523 (14)	0.0172 (2)	
C5	0.2222 (2)	0.27251 (15)	0.90312 (15)	0.0167 (2)	
H4	0.3731	0.3372	0.9097	0.020*	
C6	0.1378 (2)	0.15877 (14)	0.76997 (14)	0.0150 (2)	
C7	0.3726 (2)	0.26353 (14)	0.58419 (14)	0.0150 (2)	
H2N2	0.147 (4)	0.404 (3)	0.669 (2)	0.033 (5)*	
H1N2	0.310 (4)	0.479 (3)	0.587 (2)	0.028 (5)*	
H1N1	0.335 (4)	0.050 (3)	0.610 (2)	0.029 (5)*	

### Atomic displacement parameters (Å^2^)

**Table:**

	*U*^11^	*U*^22^	*U*^33^	*U*^12^	*U*^13^	*U*^23^
Cl1	0.02138 (16)	0.03212 (19)	0.01919 (17)	0.00492 (13)	−0.00105 (12)	−0.00194 (13)
S1	0.01841 (15)	0.01114 (14)	0.02287 (17)	0.00166 (10)	0.00657 (11)	0.00646 (11)
N1	0.0210 (5)	0.0096 (4)	0.0193 (5)	0.0014 (4)	0.0059 (4)	0.0046 (4)
N2	0.0195 (5)	0.0133 (5)	0.0271 (6)	0.0038 (4)	0.0083 (4)	0.0091 (4)
C1	0.0178 (5)	0.0168 (5)	0.0196 (6)	−0.0018 (4)	−0.0009 (4)	0.0065 (4)
C2	0.0164 (5)	0.0246 (6)	0.0246 (6)	−0.0027 (5)	0.0011 (5)	0.0107 (5)
C3	0.0167 (5)	0.0255 (6)	0.0206 (6)	0.0031 (5)	0.0034 (5)	0.0099 (5)
C4	0.0170 (5)	0.0184 (5)	0.0166 (6)	0.0042 (4)	−0.0006 (4)	0.0041 (4)
C5	0.0142 (5)	0.0148 (5)	0.0208 (6)	0.0006 (4)	0.0008 (4)	0.0049 (4)
C6	0.0160 (5)	0.0128 (5)	0.0176 (6)	0.0016 (4)	0.0021 (4)	0.0065 (4)
C7	0.0153 (5)	0.0122 (5)	0.0176 (5)	−0.0005 (4)	0.0006 (4)	0.0047 (4)

### Geometric parameters (Å, º)

**Table:**

Cl1—C4	1.7389 (13)	C1—C2	1.3948 (19)
S1—C7	1.7021 (13)	C1—H1	0.9300
N1—C7	1.3527 (15)	C2—C3	1.390 (2)
N1—C6	1.4239 (16)	C2—H2	0.9300
N1—H1N1	0.83 (2)	C3—C4	1.3918 (17)
N2—C7	1.3257 (17)	C3—H3	0.9300
N2—H2N2	0.80 (2)	C4—C5	1.3855 (18)
N2—H1N2	0.84 (2)	C5—C6	1.3968 (17)
C1—C6	1.3901 (16)	C5—H4	0.9300
			
C7—N1—C6	124.20 (11)	C4—C3—H3	120.8
C7—N1—H1N1	117.8 (14)	C5—C4—C3	121.85 (12)
C6—N1—H1N1	117.9 (14)	C5—C4—Cl1	118.15 (10)
C7—N2—H2N2	121.1 (16)	C3—C4—Cl1	119.97 (10)
C7—N2—H1N2	122.5 (14)	C4—C5—C6	118.84 (11)
H2N2—N2—H1N2	116 (2)	C4—C5—H4	120.6
C6—C1—C2	119.36 (12)	C6—C5—H4	120.6
C6—C1—H1	120.3	C1—C6—C5	120.51 (12)
C2—C1—H1	120.3	C1—C6—N1	120.46 (11)
C3—C2—C1	121.08 (12)	C5—C6—N1	118.98 (11)
C3—C2—H2	119.5	N2—C7—N1	117.85 (12)
C1—C2—H2	119.5	N2—C7—S1	121.69 (10)
C2—C3—C4	118.34 (12)	N1—C7—S1	120.45 (10)
C2—C3—H3	120.8		
			
C6—C1—C2—C3	−0.1 (2)	C2—C1—C6—N1	178.69 (12)
C1—C2—C3—C4	−1.1 (2)	C4—C5—C6—C1	−1.26 (19)
C2—C3—C4—C5	1.1 (2)	C4—C5—C6—N1	−178.74 (11)
C2—C3—C4—Cl1	−176.81 (10)	C7—N1—C6—C1	126.31 (14)
C3—C4—C5—C6	0.1 (2)	C7—N1—C6—C5	−56.21 (18)
Cl1—C4—C5—C6	177.99 (10)	C6—N1—C7—N2	−15.51 (19)
C2—C1—C6—C5	1.24 (19)	C6—N1—C7—S1	164.53 (10)

### Hydrogen-bond geometry (Å, º)

**Table:**

*D*—H···*A*	*D*—H	H···*A*	*D*···*A*	*D*—H···*A*
N2—H2*N*2···Cl1^i^	0.80 (2)	2.64 (2)	3.3583 (12)	150 (2)
N2—H1*N*2···S1^ii^	0.83 (3)	2.54 (3)	3.3619 (13)	167.5 (19)
N1—H1*N*1···S1^iii^	0.84 (2)	2.49 (3)	3.3149 (12)	167 (2)

Symmetry codes: (i) −*x*, −*y*+1, −*z*+2; (ii) −*x*+1, −*y*+1, −*z*+1; (iii) −*x*+1, −*y*, −*z*+1.
